# Nanofiber Channel Organic Electrochemical Transistors for Low‐Power Neuromorphic Computing and Wide‐Bandwidth Sensing Platforms

**DOI:** 10.1002/advs.202001544

**Published:** 2021-03-26

**Authors:** Sol‐Kyu Lee, Young Woon Cho, Jong‐Sung Lee, Young‐Ran Jung, Seung‐Hyun Oh, Jeong‐Yun Sun, SangBum Kim, Young‐Chang Joo

**Affiliations:** ^1^ Department of Materials Science & Engineering Seoul National University Seoul 151‐744 Korea

**Keywords:** nanofiber channel, neuromorphic, organic electrochemical transistors, sensors

## Abstract

Organic neuromorphic computing/sensing platforms are a promising concept for local monitoring and processing of biological signals in real time. Neuromorphic devices and sensors with low conductance for low power consumption and high conductance for low‐impedance sensing are desired. However, it has been a struggle to find materials and fabrication methods that satisfy both of these properties simultaneously in a single substrate. Here, nanofiber channels with a self‐formed ion‐blocking layer are fabricated to create organic electrochemical transistors (OECTs) that can be tailored to achieve low‐power neuromorphic computing and fast‐response sensing by transferring different amounts of electrospun nanofibers to each device. With their nanofiber architecture, the OECTs exhibit a low switching energy of 113 fJ and operate within a wide bandwidth (cut‐off frequency of 13.5 kHz), opening a new paradigm for energy‐efficient neuromorphic computing/sensing platforms in a biological environment without the leakage of personal information.

## Graphical abstract

Concepts of organic neuromorphic computing/sensing platforms for local monitoring and processing of biological signals in real time are presented. With a nanofiber architecture as a channel of a transistor, low‐power neuromorphic devices and wide‐bandwidth sensors are fabricated together on a single substrate without changing the materials, structures, and fabrication processes, demonstrating the future applications for neuromorphic computing/sensing platforms.

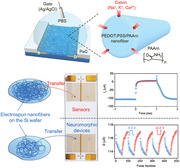

In neuromorphic computing/sensing platforms, devices can operate autonomously by sensing and processing information locally, which is essential to the development of smart healthcare technology to achieve low latency without leakage of personal information.^[^ ^1^ ^]^ Despite recent progress in organic sensors^[^ ^2^, ^3^, ^4^, ^5^, ^6^ ^]^ and neuromorphic devices,^[^ ^7^, ^8^, ^9^, ^10^ ^]^ the available devices do not sufficiently fulfill the requirements of both applications simultaneously since devices for neuromorphic computing and sensing require different channel properties. High channel conductance is suitable for low‐impedance sensors, while low channel conductance is desirable for synaptic devices capable of low‐power computing.^[^ ^7^, ^8^ ^]^ Not only these channel conductances but also the lack of appropriate methods to obtain devices with various channel properties (e.g., electrical conductivity) on a single substrate impede further developments in neuromorphic computing/sensing platforms.

Organic electrochemical transistors (OECTs)^[^ ^4^ ^]^ are promising candidates for neuromorphic computing and sensing platforms. OECTs have been applied in not only sensors^[^ ^11^, ^12^, ^13^ ^]^ but also neuromorphic devices,^[^ ^7^, ^8^, ^10^, ^14^ ^]^ such as redox synaptic transistors with a channel of (poly(3,4‐ethylenedioxythiophene) doped with poly(styrenesulfonate) (PEDOT:PSS)/poly(ethylenimine)). In OECT sensors, the gate voltage gives rise to the injection (extraction) of cations into (from) the channel and dedoping (doping) of the organic channel. OECTs employ the entire volume of their organic polymers as effective channels, leading to volumetric capacitance (*C* ^*^).^[^ ^15^ ^]^ The large capacitance enables OECTs to operate with low *V*_G_ (<1 V) and high transconductance (*g*_m_).^[^ ^16^ ^]^

However, the response speed of OECTs is usually limited by ion migration from the electrolyte to inside the channel, which is much slower than the hole drift time along the channel, making OECTs slow (≥ ms) and narrow‐bandwidth (≤ kHz) devices.^[^ ^17^, ^18^ ^]^ Recently, a microsecond response speed was reported when the ionic transit distance was minimized by embedding ions within the channel.^[^ ^19^ ^]^ However, the additional process required to form an ion membrane makes the preparation of OECTs complicated.

OECTs typically use the conducting polymer PEDOT:PSS as the channel material.^[^ ^2^, ^3^, ^4^, ^20^ ^]^ The high hole conductivity of PEDOT:PSS films is preferred in sensors due to the high signal‐to‐noise ratio, while this property is inappropriate for neuromorphic computing because a high‐conductivity channel increases energy consumption. Moreover, PEDOT:PSS itself cannot readily achieve long‐term plasticity without changes to the material^[^ ^9^, ^10^, ^21^ ^]^ and structure^[^ ^7^, ^22^ ^]^ because PEDOT:PSS‐based OECTs easily lose their state after the gate bias is disconnected owing to parasitic faradaic reactions.^[^ ^23^ ^]^ To accomplish long‐term plasticity and reduce energy consumption, a core‐sheath‐structured nanowire synaptic transistor was reported.^[^ ^22^ ^]^ The core‐sheath nanowire synaptic transistor successfully realized long‐term plasticity and reduced energy consumption to the fJ level. However, the device structure and materials of core‐sheath nanowire synaptic transistors are quite different from those of conventional OECT sensors, which makes the fabrication process complex when cofabricating OECT sensors. In addition, because the PEDOT:PSS film used for the OECT channels is mainly processed via spin coating among various formation methods of the OECT channel,^[^ ^24^, ^25^ ^]^ PEDOT:PSS is coated on the entire substrate during the spin‐coating process.^[^ ^26^ ^]^ Therefore, the spin‐coating process prevents OECTs from being fabricated with various kinds of channels, such as low‐ and high‐conductivity channels for neuromorphic computing and sensing, respectively, on a single substrate.

Here, we report electrospun PEDOT:PSS/polyacrylamide (PAAm) nanofiber channel OECTs for sensing and neuromorphic computing fabricated together on a single substrate by transferring different amounts of electrospun nanofibers onto the channels. By controlling the amount of nanofibers, the characteristics of the OECTs can be customized according to the intended application, such as low‐power neuromorphic computing and low‐impedance sensing. In dimethyl sulfoxide (DMSO)‐treated PEDOT:PSS/PAAm nanofibers,^[^ ^27^ ^]^ PAAm and PSS behave as an ion‐blocking layer and an ion injection site, respectively. Ions injected into the nanofibers through ion injection sites (i.e., PSS or pores on the surface of the nanofibers) do not instantaneously diffuse out, so some ions remain within the nanofiber. This process occurs because the PEDOT and PSS inside the nanofiber are surrounded by a naturally formed ion‐blocking layer (PAAm), which prevents ion transport, leading to long‐term plasticity. In addition, unlike the PEDOT:PSS films used in conventional OECTs, where the migration of ions in the electrolyte occurs in one direction from interfaces in contact with the electrolyte to the bottom of the channel, the nanofiber morphology enables ions to be injected from all directions from the nanofiber surface into the nanofiber, resulting in a shorter ion travel distance than that in films (**Figure** **1**b).

**Figure 1:**
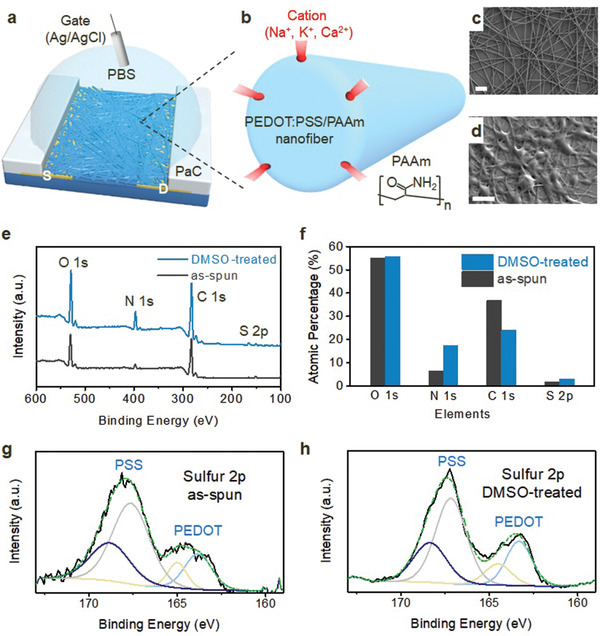
A nanofiber channel OECT and the effect of DMSO on PEDOT:PSS/PAAm nanofibers. a) Schematic of a PEDOT:PSS/PAAm nanofiber channel OECT. b) Schematic explaining the all‐directional migration of ions through the surface of a nanofiber. SEM images of c) as‐spun and d) DMSO‐treated nanofibers. Scale bar: 2 µm. e) XPS wide scan results of as‐spun and DMSO‐treated PEDOT:PSS/PAAm nanofibers used to compare the relative content of oxygen, nitrogen, carbon and sulfur. f) Comparison of atomic percentage changes in certain elements before and after DMSO treatment. XPS spectra of the S 2p peak of g) as‐spun and h) DMSO‐treated PEDOT:PSS/PAAm nanofibers.

The device fabrication process is described in detail in the Experimental Section and Figure S1 (Supporting Information). Electrospun nanofibers (Figure 1c) were mechanically transferred from a collector onto a glass substrate, and then the nanofibers were supported on a sacrificial parylene C (PaC) layer (Figure S2, Supporting Information). Interestingly, we used DMSO for not only conductivity enhancement^[^ ^28^ ^]^ but also channel patterning by dropping DMSO on the suspended nanofibers rather than adding it to the PEDOT:PSS/PAAm solution. During DMSO treatment, the transparency of the nanofibers improved (Figure S3, Supporting Information), and the surface of the nanofibers dissolved (Figure 1d and Figure S4, Supporting Information) because the high polarities of PAAm and DMSO induce strong dipole–dipole interactions.^[^ ^29^ ^]^ Afterward, the nanofibers fell into an etched channel region (Figure S1e, Supporting Information). The moderately dissolved regions of the nanofibers created by DMSO contributed to enhanced adhesion to the substrate and enabled definition of the channel after peeling off the sacrificial PaC layer (Figure S5, Supporting Information). After DMSO treatment of the PEDOT:PSS/PAAm nanofibers, the amount of PAAm and PSS increased on the surface of the nanofibers (Figure 1e–h and Note S1, Supporting Information). This architecture prevents PEDOT:PSS from disintegrating in an aqueous environment and enables long‐term plasticity and low‐energy operation since the large amount of PAAm acts as an ion‐blocking layer and PSS provides paths for easy ion transport on the surface of the nanofibers. The existence of PAAm on the nanofiber surface is the underlying mechanism of long‐term plasticity, constituting long‐term potentiation (LTP) and long‐term depression (LTD), where the conductance (i.e., synaptic weight) is increased and decreased, respectively.

A schematic of a nanofiber channel OECT is depicted in Figure 1a. A nanofiber channel OECT was fabricated with a large channel volume of 30 µL to achieve high channel conductance to compare the steady‐state and transient characteristics of film and nanofiber channel OECTs. **Figure** **2**a shows that the nanofiber channel OECT has *I*_D_ values comparable to those of the film channel OECT.^[^ ^15^, ^16^, ^17^, ^26^ ^]^ The transfer curve also shows a negligible gate leakage current over the entire operating *V*_G_ range (Figure S6a, Supporting Information). The maximum transconductance (*g*_m,max_) of 2.2 mS is attained near *V*_G_ = 0 V, which indicates that the nanofiber channel OECT exhibits large sensitivity at low *V*_G_. The output characteristics of the nanofiber channel OECT indicate clear pinch‐off and a high saturated *I*_D_ of 0.64 mA at *V*_G_ = 0 V (Figure 2b).

**Figure 2:**
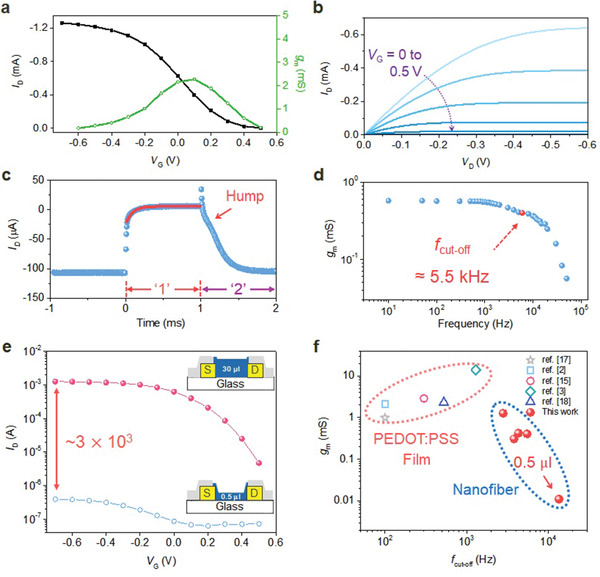
Steady‐state and transient characteristics. a) Transfer curve with associated *g*_m_ and b) output curve (*W/L* = 80 µm/2.5 µm). c) Temporal response of *I*_D_ (*W/L* = 40 µm/2.5 µm). The exponential fit of *I*_D_ is plotted, indicating time constants (*τ*) of 84 µs (region ‘1’) and 439 µs (region ‘2’). d) Frequency dependence of *g*_m_ with *f*_cut‐off,_ which is defined as a 3 dB roll‐off of the initially measured *g*_m_ in this measurement (*V*_D_ = −0.4 V, *V*_G_ = ±0.1 V). e) Transfer characteristics showing the tunable electrical conductance of the OECT with 30 µL (solid) and 0.5 µL (open) nanofiber channels (*V*_D_ = −0.4 V, *W/L* = 80 µm/2.5 µm). Inset: cross‐section schematics of nanofiber channel OECTs with 30 µL (top) and 0.5 µL (bottom) nanofibers. f) Comparison of *g*_m_ at *f*_cut‐off_ for PEDOT:PSS film and nanofiber channel OECTs.

The transient response of *I*_D_ of the nanofiber channel OECT is given in Figure 2c. Typically, the transient response of *I*_D_ in OECTs, including nanofiber channel OECTs, exhibits a monotonic decay in *I*_D_ in response to an applied *V*_G_, which means that the redistribution of ionic charge determines the response speed of OECTs; therefore, the response speed can be improved by increasing the speed of ionic migration or decreasing the travel distance of ions.^[^ ^4^ ^]^ The rise time for dedoping is 84 µs (region '1'), which is faster than that reported for conventional PEDOT:PSS film channel OECTs (>100 µs).^[^ ^2^, ^3^, ^17^ ^]^ The fast response of the nanofiber channel OECT indicates that ions can be transported from all directions through the nanofiber channel surface. The time constant for doping is 439 µs (region '2'), which is slower than that of dedoping (84 µs) (Figure 2c). Notably, a hump is observed when the injected ions revert to the electrolyte. The hump suggests that the ion‐blocking layer (PAAm) of the nanofiber channel disturbs the extraction of injected ions into the electrolyte. The ion‐blocking layer (PAAm) and the differences in resistance and capacitance of the channel between doped and dedoped states causes the discrepancies between the rise time for doping and dedoping.

The transconductance (*g*_m_) at the cut‐off frequency (*f*_cut‐off_) of the nanofiber channel OECTs is plotted in Figure 2d as a function of frequency and compared with that of PEDOT:PSS film OECTs. The nanofiber channel OECT shows a higher *f*_cut‐off_ than that of film channel OECTs but maintains a comparable *g*_m_. The *f*_cut‐off_ of film channel OECTs is governed by the channel volume as 1/2*πf*_cut‐off_ ∼ $d\sqrt{WL}$, where *d* is the film channel thickness, *W* is the channel width, and *L* is the channel length.^[^ ^15^, ^30^, ^31^ ^]^ For the nanofiber channel OECT, *W* is treated as a nominal *W*, and the cross‐sectional area of one nanofiber (*πr* ^2^) times the number of nanofibers (*n*) is proportional to *d* × *W*. By controlling the amount of nanofibers, *f*_cut‐off_ can be easily improved despite decreases in *g*_m_.

Another advantage of using nanofiber channels is that the electrical conductance and conductivity of the channel can be easily tuned by varying the amount of nanofibers (Figure S8, Supporting Information). Figure 2e shows that *I*_D_ can be tuned over three orders of magnitude by using electrospun volumes of 0.5 and 30 µL (Figure S7, Supporting Information). The tunable electrical conductance of the nanofiber channel OECTs can be understood by introducing the notion of C* and nanofiber design parameters based on the Bernards and Malliaras model (see Note S2, Supporting Information). The drain current equation of a nanofiber channel in the saturation regime is given by the following

(1) $${I_{D}}^{\mathrm{sat}}=\frac{n\left(\pi r^{2}\right)}{2L}\mu_{h}C^{*}{\left(V_{T}-V_{G}\right)}^{2}$$

where *n* is the number of nanofibers, *r* is the average radius of the nanofibers, *L* is the channel length, *μ*_h_ is the hole mobility, and *V*_T_ is a threshold voltage. According to Equation (1), controlling the number and radius of the nanofibers is an efficient method to tune the electrical conductance of the channel. We tailored the electrical conductance of the channel by varying the amount of nanofibers since the far‐field electrospinning method has difficulty controlling the radius of nanofibers due to the whipping and chaotic process. Using Equation (1), it can be approximated that the difference in the number of nanofibers within the channels shown in Figure 2e is ≈× 10^3^ between these two OECTs. The electrospun volume of 0.5 µL yields the fastest *f*_cut‐off_ in this paper; the value of 13.5 kHz is one or more orders of magnitude faster than those (usually below 1 kHz) of previously reported film channel OECTs,^[^ ^2^, ^3^, ^17^ ^]^ as shown in Figure 2f, and the corresponding transient response is 70 µs (Figure S9, Supporting Information). Although the response speed can be further improved by using a larger absolute value of *V*_G_ (Figure S10, Supporting Information), we used a peak‐to‐peak *V*_G_ of ±0.1 V to be able to measure *f*_cut‐off_ while maintaining the largest *g*_m_.

It should be noted that both a synaptic device with a low‐conductance channel for low energy consumption and a sensor with a high‐conductance channel for low impedance were manufactured simultaneously on the same substrate without changing the materials and fabrication process (Figure S2, Supporting Information). This result was achieved because we fabricated OECTs by transferring electrospun nanofibers from the collector to the desired area on the substrate rather than through spin coating.

Low switching energy is an important requirement for neuromorphic computing, especially for implantable devices where it is difficult to replace the battery and the capacity of the battery is limited. To demonstrate the low switching energy of the nanofiber channel OECT, the switching energy was calculated for an electrospun nanofiber volume of 0.5 µL by integrating the power dissipation (*P* = *I*_G_ × *V*_G_) over the pulse width, where *I*_G_ = 28.3 nA is the peak current, *V*_G_ = 10 mV is an applied gate voltage, and the pulse width is 400 µs (Figures S11 and S12, Supporting Information). The nanofiber channel OECT demonstrates a low switching energy of 113 fJ (**Figure** **3**a and Table S1, Supporting Information). This achievement is attributable to the fact that a narrow channel width on the nanometer scale offers low channel conductance, a reduced area of power dissipation, and high sensitivity due to the large surface‐to‐volume ratio that can sense small stimuli with a pulse width of tens of microseconds. Moreover, the DMSO‐treated PEDOT:PSS/PAAm nanofibers have paths (i.e., PSS) for easy ion transport on the surface.

**Figure 3:**
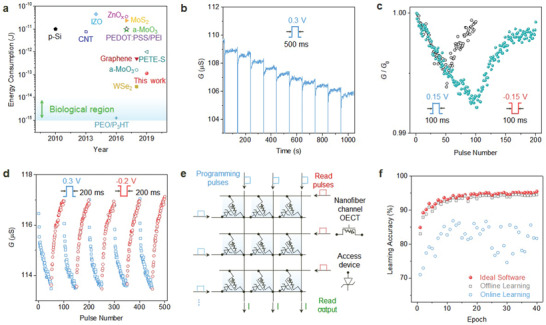
Neuromorphic behavior. a) Comparison of energy consumption associated with short‐term plasticity in other synaptic devices. b) LTD derived by applying consecutive *V*_G_ pulses (*V*_D_ = −0.2 V, pulse interval = 100 s). c) LTD and LTP with respect to pulse number (50 and 100) with symmetric pulses (*V*_D_ = −0.15 V, pulse interval = 500 ms). d) Analog channel conductance modulation under 50 repeated LTD and LTP pulses (*V*_D_ = −0.3 V, pulse interval = 300 ms). e) Circuits for nanofiber channel OECT‐ and access device‐based crossbars. f) Simulated artificial neural network accuracy for MNIST handwritten digit classification in online (blue) and offline learning (gray) for the nanofiber channel synaptic device and the ideal device accuracy (red). The simulation is performed on the basis of experimentally measured characteristics extracted from 5 depression/potentiation cycles in d).

In addition to short‐term plasticity such as paired pulse depression (Figure S13, Supporting Information), nanofiber channel OECTs can exhibit long‐term plasticity (Figure S14, Supporting Information). Figure 3b shows the LTD obtained by applying a *V*_G_ pulse with a pulse interval of 100 s repeated ten times. The channel conductance continuously decreases with the conductance difference after each *V*_G_ pulse is applied because the incomplete diffusion of injected ions out of the channel due to the disturbance of PAAm leads to repeated accumulation within the channel. LTP was also obtained with a pulse interval of 100 s (Figure S15, Supporting Information). LTD and LTP single cycling was implemented with symmetric 50 and 100 *V*_G_ pulses (Figure 3c). The cycling included consecutive positive *V*_G_ pulses for depression and then negative *V*_G_ pulses for potentiation. Although LTD and LTP cycling with 100 consecutive *V*_G_ pulses indicates a higher analog on/off ratio than that of 50 consecutive *V*_G_ pulses, the linearity degrades slightly during potentiation. To implement LTD and LTP cycling with better linearity, LTD and LTP multiple cycling was carried out with 50 consecutive *V*_G_ pulses (Figure 3d). Synaptic weight updating with linearity and symmetricity between LTD and LTP is an important element in executing highly accurate learning in neuromorphic computing. Although an almost ideal analog synaptic update was recently reported in an ionic floating‐gate memory array based on a polymer redox transistor,^[^ ^8^ ^]^ our nanofiber channel OECT demonstrates fairly linear and symmetric synaptic weight updates with the same structure, material, and fabrication process as those of the OECT designed for sensing.

To demonstrate the feasibility of the linearity and symmetricity of the nanofiber channel OECT, we simulated the Modified National Institute of Standards and Technology (MNIST) handwritten recognition data set based on backpropagation (Figure 3e,f).^[^ ^34^, ^35^ ^]^ In online learning, the multilayer perceptron (MLP) simulator takes into consideration the characteristics of the nanofiber channel OECT in training with 60 000 images randomly picked from a training dataset and classifying 10 000 images from a testing dataset.^[^ ^36^ ^]^ In offline learning, the MLP simulator only performs the classification using the nanofiber channel OECT with a neural network pretrained by software. Our nanofiber channel OECT exhibits high classification accuracy, approaching 94.5% in offline learning and 86.9% in online learning, where the algorithm limit of the software is 95.5% (Figure 3f). The classification accuracy in online learning deviates by less than 10% from the ideal case. Recently, an ionic floating‐gate memory array and two‐terminal memristor based on Ag migration in the etched region showed higher accuracy than our device. Nevertheless, the nanofiber channel OECT presented better classification accuracy than that reported in resistive memories (20–70%) and phase change memories (82%).^[^ ^37^, ^38^, ^39^ ^]^

In conclusion, we have demonstrated nanofiber channel OECTs for neuromorphic computing and sensing, which were fabricated together on a single substrate without changing the materials, structures, and fabrication processes. The electrical conductance of the channel could be easily tailored by up to ≈10^3^ times for the desired application by controlling the amount of nanofibers. By DMSO treatment of the PEDOT:PSS/PAAm nanofibers, long‐term plasticity was realized because a large amount of PAAm moved to the nanofiber surface and acted as an ion‐blocking layer. By virtue of the nanofiber architecture, our OECT exhibited not only a high cut‐off frequency of 13.5 kHz but also an ultralow energy consumption of 113 fJ. These results will potentially open up a path toward smart healthcare platforms consisting of neuromorphic devices and sensors for interfacing with biology.

## Experimental Section

##### Nanofiber Preparation

The nanofibers consisted of a conducting polymer (PEDOT:PSS) and an insulating polymer (PAAm), which was prepared following a previously reported method.^[^ ^27^ ^]^ Due to the high molecular weight and long polymer chain of PAAm, PEDOT:PSS stably operates in an aqueous environment without cross‐linkers. Before electrospinning, the PEDOT:PSS needs to be blended with PAAm, which is used as a carrier in the electrospinning process. First, PEDOT:PSS and PAAm solutions were separately prepared. As‐received PEDOT:PSS solution (Heraeus, Clevios PH 1000) was filtered via a 0.45 µm syringe filter and frozen in a freezer for a day at −20 °C. Then, the frozen solution was fully dried for 3 days at 5 mTorr vacuum in a freeze dryer (FD5508, IlShinBioBase). A 0.17 g mass of the obtained PEDOT:PSS solute was dissolved in 9 mL deionized (DI) water. Then, the mixture was stirred for 24 h at room temperature at 300 rpm. PAAm solution was prepared by adding 0.68 g (9.57 mmol) acrylamide (Sigma Aldrich), 40 µL (8 µmol) ammonium persulfate (Sigma Aldrich) as an initiator, and 3.4 µL *N*,*N*,*N*`,*N*`‐tetramethylethylenediamine (Sigma Aldrich) as an accelerator into 9 mL DI water. The mixture was stirred for 2 h at 70 °C and 300 rpm. After polymerization of the acrylamide, 7.5 mL PAAm solution was poured into the PEDOT:PSS solution prepared from the freeze‐dried solute, and the mixture was stirred for 24 h at room temperature and 300 rpm to obtain sufficient homogeneity. It should be mentioned that dimethyl sulfoxide (DMSO) was not added here because DMSO was later exploited for channel patterning during device fabrication, as well as conductivity improvement. The synthesized PEDOT:PSS/PAAm solution had a PEDOT:PSS to PAAm ratio of 25 wt%.

For electrospinning (NanoNC, EP100), the applied direct current voltage was 6 kV, the flow rate was 0.16 mL h^−1^ with a nozzle of 5 mm in diameter, the tip‐to‐collector (Si wafer) distance was 7.5 cm, the relative humidity was 15%, and the temperature was 25 °C. The volume of electrospun nanofibers used was 30 µL, except for the sample used to calculate energy consumption and the fastest response speed, which was fabricated with a volume of 0.5 µL. The electrospun nanofibers were annealed for crystallization in a dry oven for 24 h at 120 °C.

##### Device Fabrication

The device fabrication process is basically based on a previously reported method applied before electrospinning.^[^ ^16^ ^]^ Glass slides (26 mm × 76 mm) used as substrates were cleaned with soap, followed by sonication in acetone, isopropyl alcohol, and DI water. Chrome (10 nm) and gold (100 nm) were evaporated using an e‐beam evaporator onto photolithographically patterned photoresist (AZ 5214E, 3800 rpm) using a contact aligner. The metal interconnects were defined through lift‐off in acetone. To insulate the interconnects from the electrolyte, 1.5 µm of parylene C (PaC) was deposited with A‐174 Silane acting as an adhesion promoter. A dilute soap solution (1%) of a Micro‐90 industrial cleaner was spin coated onto the 1st PaC layer, and 2nd PaC layer of 2.5 µm was subsequently deposited. The 2nd PaC layer served later as a sacrificial layer and was used to define a channel area by peeling from the substrate. The sample was then patterned with a photoresist (AZ5214E, 2000 rpm), which was thicker than the photoresist used for interconnect patterning. The patterned areas were etched with reactive ion etching. The electrospun PEDOT:PSS/PAAm nanofibers were mechanically transferred from the p‐Si wafer to the glass substrate, where the nanofibers were supported on the 2nd PaC layer. Posttreatment was performed by dropping 4 vol% DMSO onto the nanofibers, and the nanofibers were moderately dissolved. The dissolved area contributed to adherence to the substrate during baking at 80 °C for 5 min in a dry oven. The channel was patterned by peeling off the 2nd PaC layer using scotch tape, followed by baking the sample again at 120 °C for 2 h in a dry oven to remove solvents in the nanofibers. The sample was immersed in DI water for 6 h to remove unwanted residues and excess low‐molecular‐weight compounds. A polydimethylsiloxane well was placed around the channel to define the electrolyte reservoir.

##### Electrical Characterization

Electrical characterizations were carried out in phosphate‐buffered saline (PBS) solution using Ag/AgCl pellets (Warner Instruments) with a Keithley 4200A‐SCS. The analyses provided the OECT transfer (sweeping *V*_G_ from −0.7 to 0.5 V with *V*_D_ = −0.4 V) and output (sweeping *V*_D_ from 0 to −0.6 V and stepping *V*_G_ from 0 to 0.5 V with a step of 0.1 V) characteristics. The response time was calculated for dedoping with a time constant of an exponential fit and for doping with the time needed to reach 90% of the saturation current to avoid the effect of the hump (Figure 2c) during measurement.

##### Material Characterization

The morphology of the electrospun nanofibers and the channel area of the fabricated device were observed using field emission scanning electron microscopy (MERLIN, Compact) after platinum coating. Ultraviolet–visible spectra were obtained by UV/Vis spectroscopy (V‐770, JASCO). A background spectrum of a bare cover glass (18 mm × 18 mm, MARIENFELD) was recorded for baseline correction. Samples were prepared by transferring 10 and 50 µL electrospun nanofibers onto a cover glass, treatment with 4% DMSO v/v, and drying for 2 h at 120 °C in a dry oven. Atomic force microscopy (AFM) imaging was performed in noncontact or tapping mode by scanning the sample surface using an NX‐10 (Park Systems). X‐ray photoelectron spectroscopy (XPS) measurements were carried out using an AXIS‐HSi (Kratos, UK) system. The XPS spectra were fitted by using CasaXPS software.

##### Simulation

The simulation was implemented on the basis of the platform “+NeuroSim.”^[^ ^32^, ^33^, ^34^ ^]^ For the simulation, a three‐layer network including one hidden layer was used with 400 input neurons corresponding to a 20 × 20 MNIST image, 100 hidden neurons, and 10 output neurons corresponding to 10 classes of digits (0–9). The nonlinearity, cycle‐to‐cycle variation, on/off ratio, number of conductance states, and wire resistance of 78 Ω, which was measured from the device, were fed into the simulation.

## Conflict of Interest

The authors declare no conflict of interest.

## Author Contributions

S.‐K.L., S.B.K., and Y.‐C.J. conceived the idea and designed the research. S.‐K.L. fabricated devices and performed measurements. S.‐H.O. helped to fabricate devices. J.S.L. and Y.‐R.J. prepared PDMS well and PEDOT:PSS/PAAm solution, respectively. Y.C. and S.B.K. verified the synaptic devices through MNIST simulation. J.‐Y.S., S.B.K., and Y.‐C.J. supervised the project and reviewed and commented on the manuscript.

## Supporting information



Supporting Information
