# Supplementary material for: Nanofiber Channel Organic Electrochemical Transistors for Low‐Power Neuromorphic Computing and Wide‐Bandwidth Sensing Platforms
Source: Adv Sci (Weinh). 2021 Mar 26;8(10):2001544. doi: 10.1002/advs.202001544 (PMC8132164; doi:10.1002/advs.202001544)
Supplement: Supplementary file 1 — Supporting Information [file ADVS-8-2001544-s001.pdf]

© 2020 Wiley-VCH GmbH

ADVANCED SCIENCE
Open Access

Supporting Information

for *Adv. Sci.*, DOI: 10.1002/advs.202001544

Nanofiber Channel Organic Electrochemical Transistors for Low-Power Neuromorphic Computing and Wide-Bandwidth Sensing Platforms

Sol-Kyu Lee,[1] Young Woon Cho[1], Jong-Sung Lee[1], Young-Ran Jung[1], Seung-Hyun Oh[1], Jeong-Yun Sun[1], and SangBum Kim[1] *, and Young-Chang Joo[1] **

Nanofiber Channel Organic Electrochemical Transistors for Low-Power Neuromorphic Computing and Wide-Bandwidth Sensing Platforms

Sol-Kyu Lee,[1] Young Woon Cho[1], Jong-Sung Lee[1], Young-Ran Jung[1], Seung-Hyun Oh[1], Jeong-Yun Sun[1]*, and SangBum Kim[1] *, and Young-Chang Joo[1] *

[1]*Department of Materials Science & Engineering, Seoul National University, Seoul 151-744, Korea*

***Corresponding authors:**

Jeong-Yun Sun*

E-mail: jysun@snu.ac.kr, Tel: +82-2-880-1714

1 Gwanak-ro, Gwanak-gu, Seoul 151-744, Korea

SangBum Kim*

E-mail: sangbum.kim@snu.ac.kr, Tel: +82-2-880-7359

1 Gwanak-ro, Gwanak-gu, Seoul 151-744, Korea

Young-Chang Joo*

E-mail: ycjoo@snu.ac.kr, Tel: +82-2-880-8986, Fax: +82-2-883-8197

1 Gwanak-ro, Gwanak-gu, Seoul 151-744, Korea

Supplementary Notes

Supplementary Note 1- Composition analysis of PEDOT:PSS/PAAm nanofibers with respect to position

The chemical composition of PEDOT:PSS and PAAm on the nanofiber surface was explored using XPS analysis. Nitrogen atoms are contained only in PAAm, whereas sulfur atoms are included in PEDOT and PSS. Therefore, the ratio of N 1s to S 2p can be estimated by integrating the area ratio of the peaks corresponding to PAAm to those corresponding to PEDOT:PSS. The ratio of PAAm to PEDOT:PSS increased from 4.06:1 in the as-spun nanofibers to 5.97:1 after DMSO treatment (Figure 1e,f). The higher PAAm ratio originates from the larger content of PAAm than PEDOT:PSS in the nanofibers. After DMSO treatment, the PAAm content increases by 47% on the surface of the nanofibers compared to PEDOT:PSS. Consequently, the surface of the PEDOT:PSS/PAAm nanofiber has a higher content of PAAm than PEDOT:PSS, and the ratio increases after DMSO treatment.

Sulfur atoms are contained in both PEDOT and PSS; the sulfur atoms are included within the thiophene ring in PEDOT and in the sulfonate moiety in PSS[1,2]. To further understand the component changes after DMSO treatment, the ratios of PEDOT and PSS must be analyzed separately. The S 2p peaks occur at two binding energies (Figure 1g,h). The lower binding energy peak between 163.5 and 164.5 eV corresponds to the sulfur atoms in PEDOT[1,2]. The higher binding energy peak near 167.5 eV corresponds to the sulfur atoms in PSS[2,3]. The estimated area ratios of PEDOT to PSS are 1:2.32 and 1:3.07 for as-spun and DMSO-treated nanofibers, respectively. The PSS content is increased by 33% on the surface of the nanofibers after DMSO treatment, while PEDOT moves further inside and is more densely packed in the nanofibers. Therefore, the PEDOT:PSS/PAAm nanofibers have PEDOT-rich cores and excess PSS and PAAm in the outer region. This composition change is related to PAAm and PSS being hydrophilic and PEDOT being hydrophobic, causing some amount of PAAm and PSS to be attracted to the surface during DMSO treatment due to the high polarities of these two compounds and DMSO.

Supplementary Note 2- Model of nanofiber channel OECT operation

We introduce here the drain current equation for nanofiber channel OECTs with the volumetric capacitance (C^*) based on the model by Bernards and Malliaras[4]. The Bernards and Malliaras model starts with Ohm's law applied to the channel and the following assumption in the channel:

$$J = \sigma \cdot \left[\frac{dV(x)}{dx}\right] \quad (1.1)$$

where J is the electric current density via the channel, and σ is channel conductivity, given by the following:

$$\sigma = q \cdot \mu_h \cdot p(x) \quad (1.2)$$

$$I_D = n \cdot (\pi r^2) \cdot q \cdot \mu_h \cdot p(x) \cdot \left[\frac{dV(x)}{dx}\right] \quad (1.3)$$

where n is the number of nanofibers, r is the average radius of the nanofibers, μ_h is the hole mobility, and $p(x)$ is the hole density, given by the following:

$$p(x) = SO_3^- - Q^+(x) \quad (1.4)$$

where SO_3^- is the density of sulfonate groups that are compensated for by holes in PEDOT:PSS, and $Q^+(x)$ is the density of cations that enter the channel when a positive V_G is applied to the electrolyte. The channel is treated as a volumetric capacitor, giving the following:

$$Q^+(x) = \frac{C^*}{q} \cdot [V_G - V(x)] \quad (1.5)$$

Substituting equations (1.4) and (1.5) into (1.3), we obtain an equation for I_D:

$$I_D = n \cdot (\pi r^2) \cdot q \cdot \mu_h \cdot \{SO_3^- - Q^+(x)\} \cdot \left[\frac{dV(x)}{dx}\right] \quad (1.6)$$

$$I_D = n \cdot (\pi r^2) \cdot q \cdot \mu_h \cdot \left\{SO_3^- - \frac{C^*}{q} \cdot [V_G - V(x)]\right\} \cdot \left[\frac{dV(x)}{dx}\right] \quad (1.7)$$

Because the steady-state current density throughout the OECT channel is constant, integrating equation (1.7) over the length of the channel yields a straightforward current-voltage relationship.

$$\int_0^L I_D\,dx = \int_0^{V_D} n \cdot (\pi r^2) \cdot q \cdot \mu_h \cdot \left\{SO_3^- - \frac{C^*}{q} \cdot [V_G - V(x)]\right\} dV \quad (1.8)$$

$$I_D \cdot L = n \cdot (\pi r^2) \cdot \mu_h \cdot C^* \cdot \left(\frac{q \cdot SO_3^- \cdot V_D}{C^*} - V_G V_D + \frac{1}{2} V_D^2\right) \quad (1.9)$$

$$I_D \cdot L = n \cdot (\pi r^2) \cdot \mu_h \cdot C^* \cdot \left(\frac{q \cdot SO_3^-}{C^*} - V_G + \frac{1}{2} V_D\right) V_D \quad (1.10)$$

where $V_T = \frac{q \cdot SO_3^-}{C^*}$

$$I_D = \frac{n \cdot (\pi r^2)}{L} \cdot \mu_h \cdot C^* \cdot \left(V_T - V_G + \frac{1}{2} V_D\right) V_D \quad (1.11)$$

In the saturation regime (i.e., $V_D > V_G - V_T$), the current and transconductance are given by the following:

$$I_D^{sat} = \frac{n \cdot (\pi r^2)}{2L} \cdot \mu_h \cdot C^* \cdot (V_T - V_G)^2 \quad (1.12)$$

$$g_m^{sat} = \frac{n \cdot (\pi r^2)}{L} \cdot \mu_h \cdot C^* \cdot (V_T - V_G) \quad (1.13)$$

Fabrication process of the nanofiber channel OECT

Nanofiber channel OECTs require fabrication methods that have not been used in film channel OECTs. This is because the nanofibers are suspended on a second PaC layer rather than adhered to the substrate within a channel after the transfer process, as shown in Figure S1d. In this state, channels cannot be defined since the nanofibers are peeled off together with the sacrificial second PaC layer during the peel-off process. Therefore, a novel method for attaching nanofibers to the substrate is needed to obtain channels.

To fabricate a nanofiber channel OECT, we used DMSO for not only improving the conductivity of the nanofiber but also channel patterning. DMSO is usually used to increase the conductivity of PEDOT:PSS by mixing with the PEDOT:PSS dispersion. DMSO was dropped on the nanofiber dissolved PAAm at the nanofiber surface, and then the channel was filled with nanofibers, as demonstrated in Figure S1e. If either excessive DMSO is dropped on a nanofiber or DMSO treatment is implemented on an as-spun nanofiber without crystallization annealing, the architecture of the nanofiber will collapse. Under a limited DMSO content, the morphology of the nanofiber will remain intact, but the nanofiber will not dissolve. The nanofiber will then remain suspended on the second PaC layer rather than being attached to the substrate, hence disabling channel patterning. Thus, controlling the DMSO content and moderating the dissolution of nanofibers are important for the fabrication of nanofiber channel OECTs.

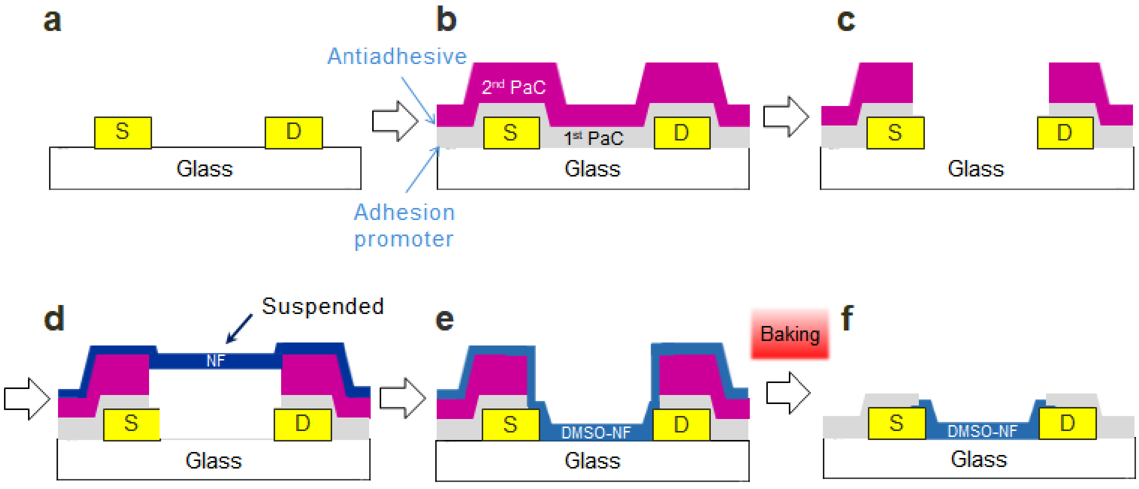

Figure S1. Fabrication steps. a) Deposition and patterning of source/drain electrodes on the glass slide substrate. b) Application of the adhesion promoter to the surface and deposition of the first PaC layer. After that, a light antiadhesive layer is formed on the

first PaC layer, and then a second PaC layer is deposited. c) Channel photolithography and reactive ion etching. d) Electrospun nanofibers transferred onto the channel region. The nanofibers are suspended on the second PaC layer. e) Dropping DMSO on the nanofibers. The surface of the nanofibers dissolves when DMSO is dropped, and the nanofibers fall into the etched area. The nanofibers in the etched area adhere to the substrate during baking. f) Peeling off the sacrificial second PaC layer with nanofibers outside the channel region to define the channel.

Transfer method of electrospun nanofibers

Typically, channel formation in OECTs is accomplished via the spin-coating process. Spin coating allows not only channel formation on a whole substrate at a time but also determination of the channel thickness by controlling the rotation speed of the chuck and the number of spins. During spin coating, a polymer solution is coated onto the entire exposed surface of a substrate, which enables the fabrication of OECTs with the same channel material and thickness in a single batch. To make different channel materials or thicknesses on the substrate, a further photolithography process is needed.

However, the solvents used in conventional photolithography, such as developer and photoresist stripper, have deleterious effects, including delamination and swelling, on organic films. Therefore, the formation of various kinds of channels is challenging in film channel OECTs processed via spin coating. This challenge makes it difficult to fabricate neuromorphic devices with low-conductivity channels and sensors with high-conductivity channels on a single substrate.

For nanofiber channel OECTs, various amounts of nanofibers are separately electrospun on each collector (e.g., Si wafer) (Figure S2a,b) and can be transferred to the desired area. The transfer method endows nanofiber channel OECTs with various amounts and kinds of nanofibers. By fabricating a nanofiber channel OECT with small and large amounts of nanofibers on the substrate, we achieved neuromorphic devices and sensors on a single substrate.

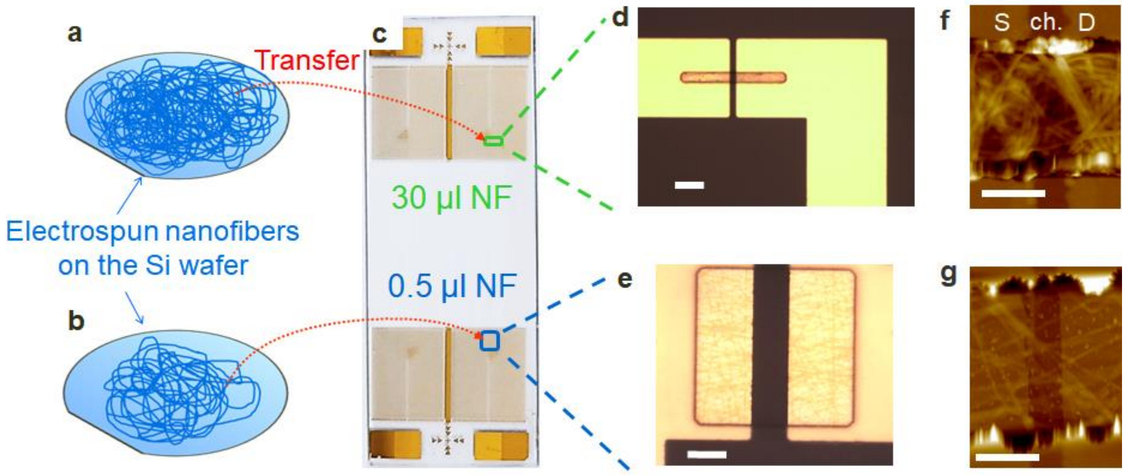

Figure S2. Fabricating a nanofiber channel OECT with both small and large amounts of nanofibers on a glass slide using the transfer method. Schematics of electrospun nanofibers with a volume of (a) 30 μl and (b) 0.5 μl. c) Photograph of a patterned glass

slide to be transferred. Optical microscopy and AFM images of fabricated devices with nanofiber volumes of (d, f) 30 µl and (e, g) 0.5 µl. Scale bar: 10 µm.

Transparency of PEDOT:PSS/PAAm nanofibers

The transparency of PEDOT:PSS/PAAm nanofibers varies with regard to the amount of nanofibers. The photograph in (b) of a bare cover glass shows transparency, while (c) shows that the color of DMSO-treated PEDOT:PSS/PAAm nanofibers is light blue, indicating the incorporation of increased amounts of PEDOT:PSS into the nanofibers. The sample with 10 µl of nanofibers exhibits a transmittance above 90% even before DMSO treatment. The sample with 50 µl of nanofibers and without DMSO exhibits less than 80% transmittance in the 300-900 nm wavelength region. After DMSO treatment, the transmittance significantly improved, reaching more than 90% in the 300-600 nm wavelength region and 80% or more in the 600-900 nm wavelength region.

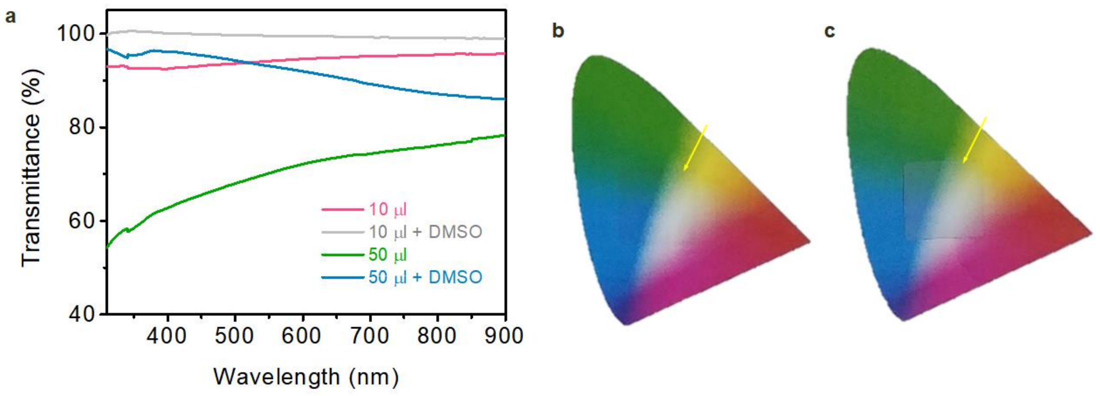

Figure S3. UV-vis spectra and photographs of PEDOT:PSS/PAAm nanofibers coated on cover glasses. a) Transmittance of PEDOT:PSS/PAAm nanofibers with respect to the amount of nanofibers (10 µl and 50 µl) before and after DMSO treatment. Photographs of (b) a bare cover glass and (c) a cover glass coated with 50 µl of nanofibers. The cover glass position is represented by the yellow arrows.

AFM images

The morphology of DMSO-treated PEDOT:PSS/PAAm nanofibers and the dissolution of a joint at the cross junction between two nanofibers were confirmed by AFM. The nanofibers appeared to be melted and connected at the cross junction and adhered to substrates. The heights of the two nanofibers were 53 (region 1) and 39 nm (region 2), respectively, and the height of the cross junction of the two nanofibers was 83 nm (region 3), demonstrating that the nanofibers were moderately melted and connected rather than simply touching. This morphology allows channels to adhere well to the substrate.

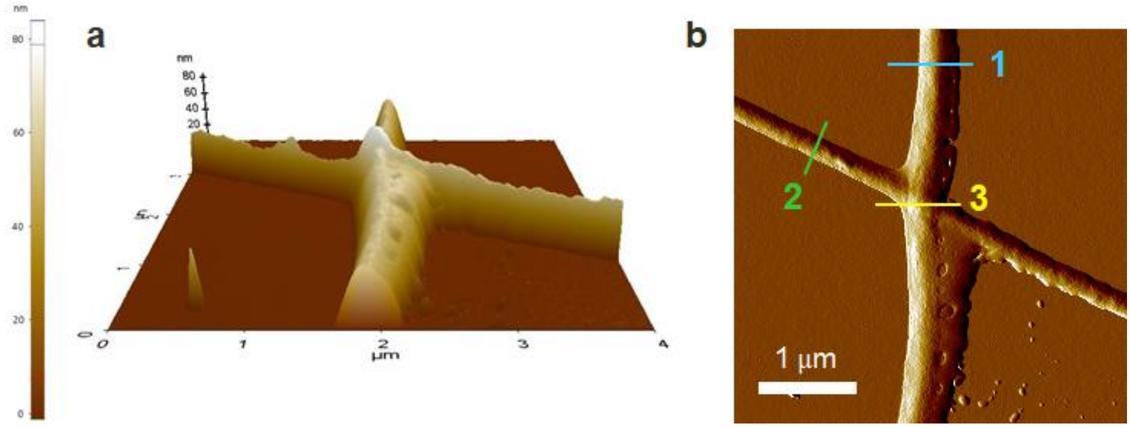

Figure S4. Surface morphology of DMSO-treated electrospun nanofibers. Tapping mode AFM images of DMSO-treated PEDOT:PSS/PAAm nanofibers (a) Height and (b) amplitude images.

SEM images

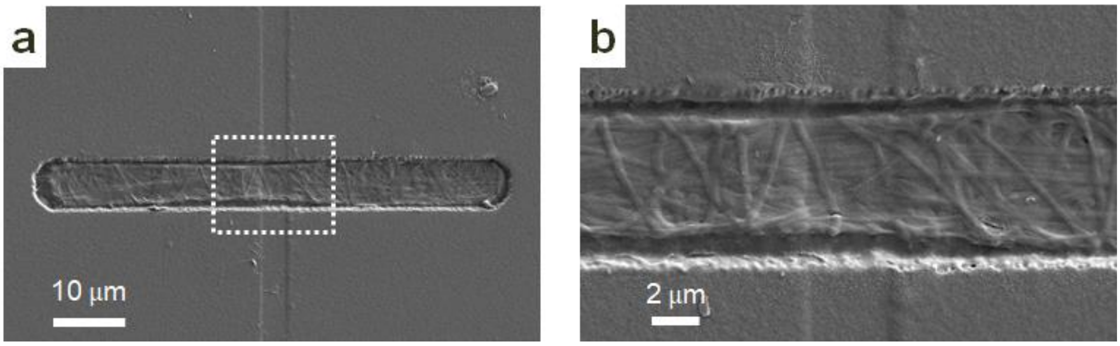

Figure S5. SEM images of a fabricated nanofiber channel OECT. a) The channel comprising DMSO-treated PEDOT:PSS/PAAm nanofibers defined after peeling off the second PaC layer; b) zoomed-in image (*W*/*L* = 5 µm/5 µm).

Gate leakage current

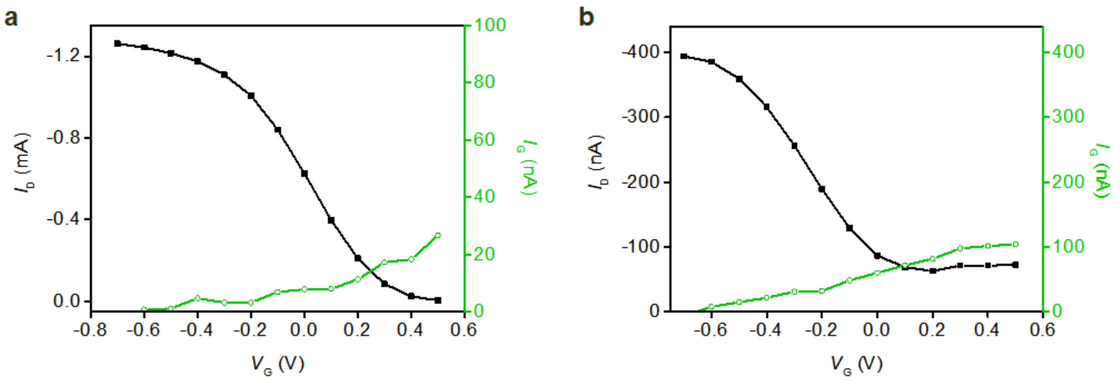

Figure S6. Transfer curves with gate leakage current. a) The largest gate leakage current of a 30 µl nanofiber channel in this graph is 26 nA, and the corresponding I_D is -4.7 µA at V_G = 0.5 V. Hence, the gate leakage current is negligible over the entire operating gate voltage range. b) The gate leakage current of a 0.5 µl nanofiber channel (V_D = -0.4 V, W/L = 80 µm/2.5 µm)

Tunable transfer characteristics

The channel conductance of nanofiber channel OECTs is easily tuned by changing the amount of nanofibers. A larger amount of nanofibers results in both a higher maximum drain current and higher $g_{m,max}$. $V_G(g_{m,max})$ shifts from a negative V_G to a positive V_G with an increasing amount of nanofibers.

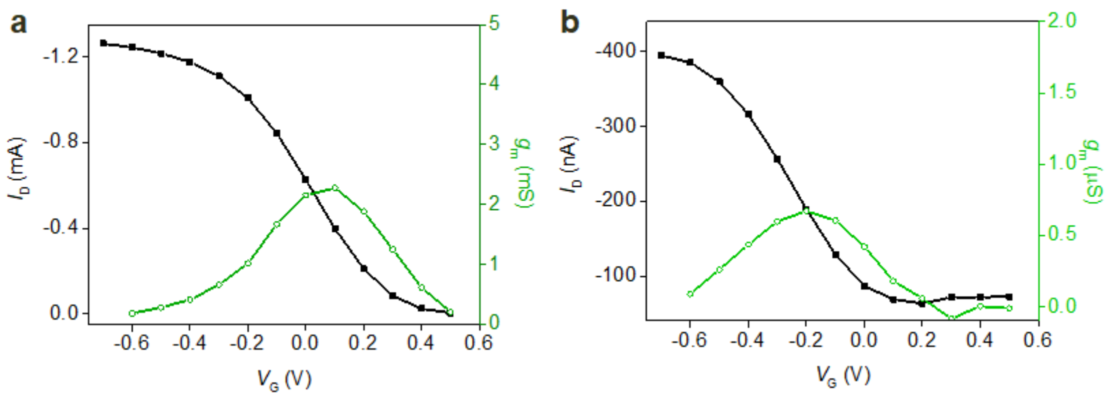

Figure S7. Transfer curves with large and small amounts of nanofibers in the channel. (a) High-conductance and (b) low-conductance channels consisting of 30 µl and 0.5 µl nanofibers, respectively. The on/off current ratios for high and low channel conductance are 268 and 5, respectively.

Tunable channel conductivities

Channel conductance can be easily controlled on a single substrate, even by spin coating. However, this approach degrades spatial resolution for application such as EEGs (electroencephalograms) or ECGs (electrocardiograms) because OECTs with large channels are needed to obtain large conductance and signal-to-noise ratio. Therefore, control of channel conductivity is preferred to channel conductance.

To demonstrate that nanofiber channel OECTs with various channel conductivities in same channel sizes can be fabricated on a single substrate, the devices were fabricated by adjusting the PEDOT:PSS to PAAm ratio on a single substrate, and their electrical characteristics were explored. The synthesized PEDOT:PSS/PAAm solution had a PEDOT:PSS to PAAm ratio of 15, 20, and 25 wt%. As expected, the on-current and g_m increased with increasing the ratio of PEDOT:PSS to PAAm. It was confirmed that our nanofiber channel OECTs with various channel conductivities can be fabricated with same channel sizes by adjusting the PEDOT:PSS to PAAm ratio.

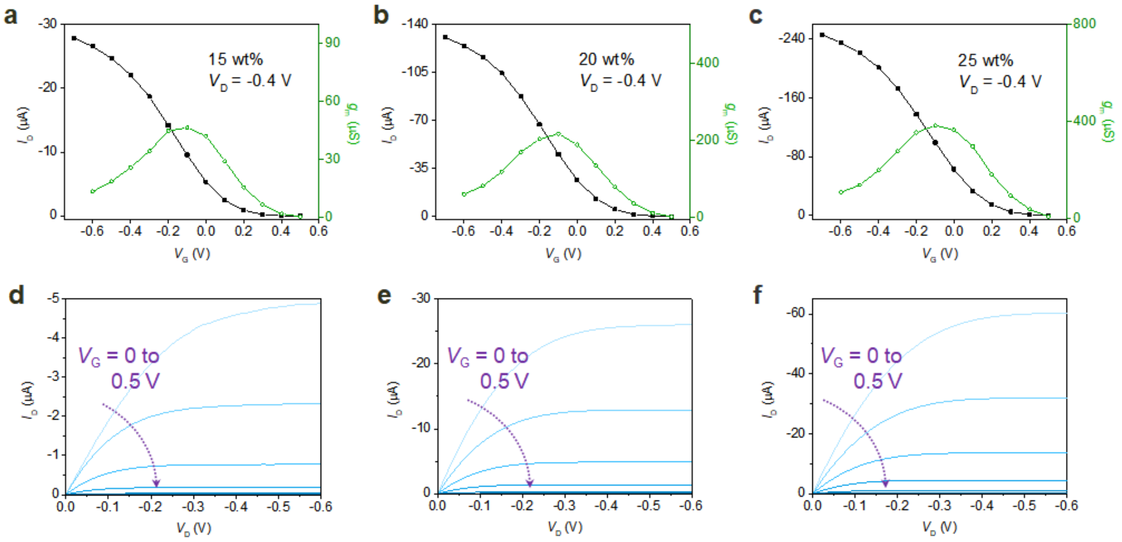

Figure S8. Transfer and output curves with different channel conductivities in the same channel size. Transfer curves for the composition of PEDOT:PSS to PAAm with (a) 15, (b) 20, and (c) 25 wt%. Output curves for the composition of PEDOT:PSS to PAAm with (d) 15, (e) 20, and (f) 25 wt%. (W/L = 10 μm/10 μm).

Fastest response of the nanofiber channel OECT

The electrospun volume of 0.5 µl yields the fastest $f_{cut\text{-}off}$ in this paper; the value of 13.5 kHz. Even if g_m is small, devices with high $f_{cut\text{-}off}$ (i.e., response speed) could be optimal building blocks for integrated bioelectronics, such as digital logic gates (e.g., NAND, NOR)[20].

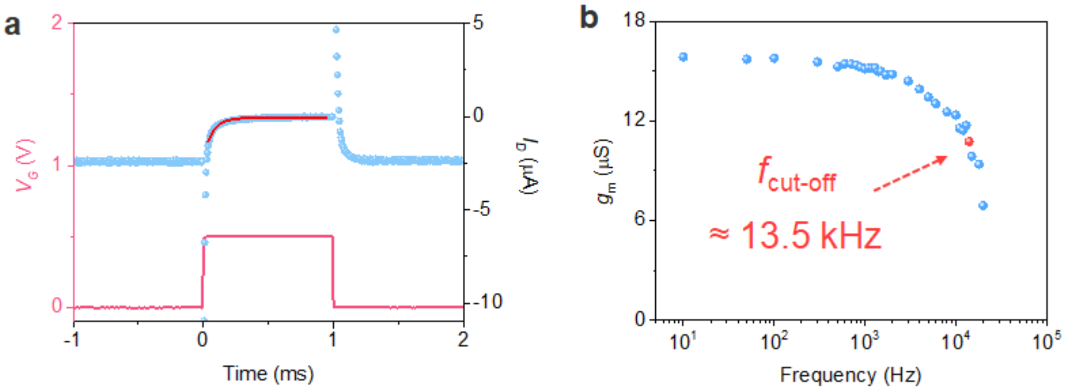

Figure S9. Fastest temporal response of the drain current. a) The exponential fit of I_D is plotted, corresponding to a time constant (τ) of 70 µs (W/L = 10 µm/10 µm). b) Frequency dependence of g_m with $f_{cut\text{-}off,}$ which is defined as a 3 dB roll-off of the initially measured g_m in this measurement (V_D = -0.4 V, V_G = ±0.1 V).

Variation in response time with respect to gate voltage

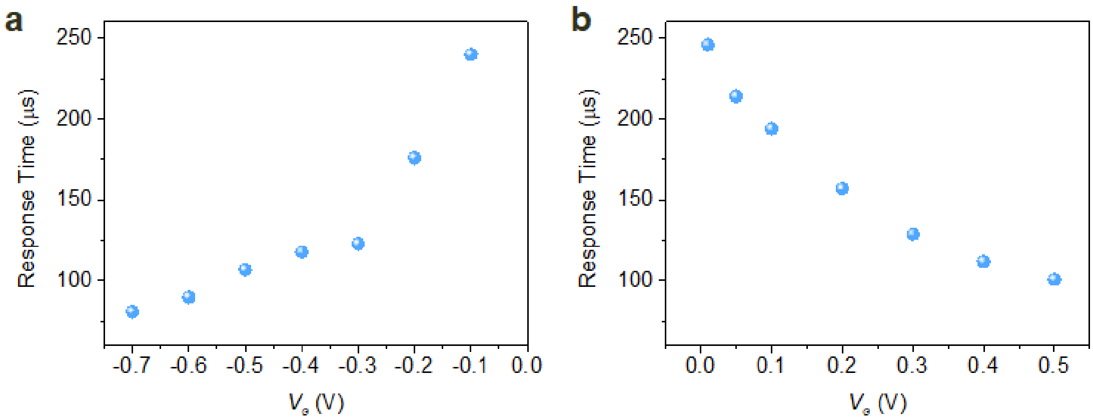

Figure S10. Response time with respect to negative and positive V_G values. The response time decreases with increasing absolute value of V_G. This behavior means that the tendency of (a) extracting or (b) injecting cations from or into the channel, respectively, becomes stronger at larger V_G (W/L = 10 µm/5 µm).

.

Calculation of switching energy

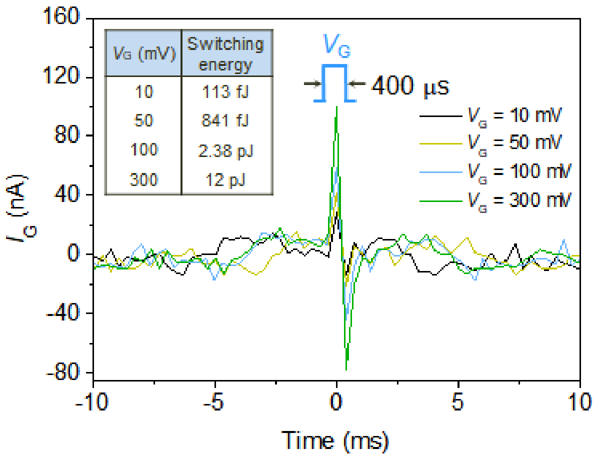

Figure S11. Switching energy consumption. Spikes were triggered by various V_G to minimize switching (V_D = -2 mV, W/L = 2 µm/5 µm). The minimized switching energy of 113 fJ was obtained with V_G = 10 mV. The average leakage I_G was 0.42 nA, which is negligible to the peak current of 28.3 nA.

Calculation of signal-to-noise ratio

The signal-to-noise ratio (SNR) was measured to show the reliability of the results of calculation of switching energy. The SNR was estimated by the results of V_G = 10 mV from Figure S11, and the equation is as follows:

$$SNR = \frac{P_S}{P_N} = \frac{{r_S}^2}{{\sigma_N}^2}$$

where P_S is the power of a signal, P_N is the power of background noise, r_s is the distance from the signal peak to the average leakage current (I_{avg}) baseline, and ${\sigma_N}^2$ is the variance of the noise defined as ${\sigma_N}^2 = \frac{1}{N}\sum(I_N - I_{avg})^2$. The signal amplitude ($r_s$) and 76 points of baseline I_G were selected for the calculation of their variation. The measured SNR was 1.6×10^1, which can be converted to 12 dB from the following relationship:

$$SNR\ (dB) = 10log_{10}\frac{P_S}{P_N}$$

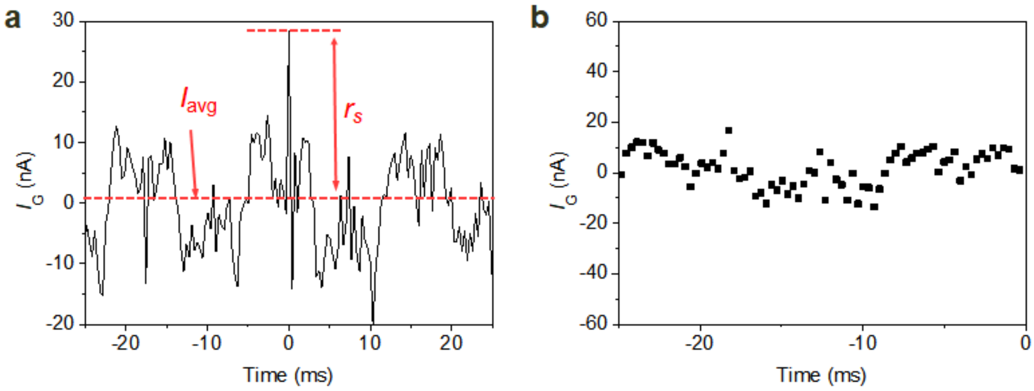

Figure S12. Calculation of SNR of the data when measuring switching energy. a) A spike was triggered by V_G = 10 mV from Figure S11. I_{avg} was used as baseline for the determination of r_S. b) Leakage current points were selected from (a) for the calculation of standard deviation.

Emulation of biological synapses

In biological synapses, the paired-pulse depression (PPD) phenomenon is observed when two spikes are transmitted to a postsynaptic neuron with a narrow time interval between spikes. As the interspike interval increases, the influence of the first spike on the second spike decreases, and eventually, any causality between the two spikes disappears. To establish this functionality in the nanofiber channel OECT, a long channel (L = 5 mm) is adopted to exhibit spike-and-recover behavior wherein hole transport in the channel, rather than ion migration, is the limiting step[16,17]. A pair of gate pulses is applied, and the ratio of PPD exponentially decreases with increasing interspike timing (Δt). When Δt is sufficiently large (> 250 ms), the cations that are injected into the channel during the first pulse have enough time to diffuse back to the electrolyte before the second pulse arrives. This trend is well fitted (R^2 = 0.99) by a double exponential decay function with two time constants, τ_1 = 1.6 ms and τ_2 = 38.2 ms, which are slightly faster values than those measured in biological[18] and artificial synapses[12]. These smaller time constants contribute to the reduced migration length of ions for nanofiber channels than film channels. This fast synaptic functionality is appealing for the development of neurorobotics and neuroprosthetics, such as artificial afferent nerves[19].

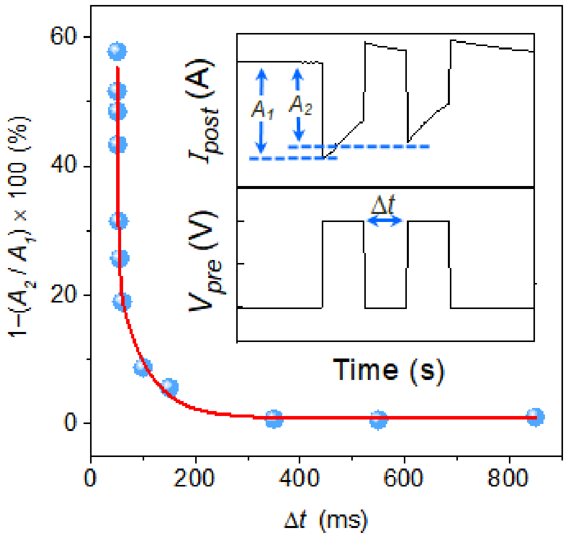

Figure S13. PPD, defined as the ratio of the amplitude changes between the first spike (A_1) and the second spike (A_2) (V_G = 0.5 V, V_D = -0.1 V, pulse width = 50 ms, W/L = 10 mm/5 mm).

Short-term and long-term plasticity

After successive positive V_G pulses, relaxation of conductance occurs because cations diffuse back spontaneously to the electrolyte. However, the change in conductance is stabilized at the conductance gap from the initial current in the case of pulse widths of 20, 50, and 100 ms, implying long-term plasticity. The conductance gap increases with a longer pulse width because more ions are injected into the channel and rest within the channel. A short pulse width of 10 ms shows a nonpermanent change in synaptic weight, resulting in short-term depression.

The precise pulse width that causes the transition from short-term plasticity to long-term plasticity is difficult to define because the transition gradually occurred, not sharply at some point, by various conditions such as the magnitude of V_G, pulse width, pulse interval, and the number of pulses. Larger V_G and/or pulse width and/or shorter pulse interval and/or more the number of pulses inject more ions into deeper nanofibers inside, followed by more ions resting inside nanofibers, causing the changed state to slowly recover to an initial state. This phenomenon continuously occurs.

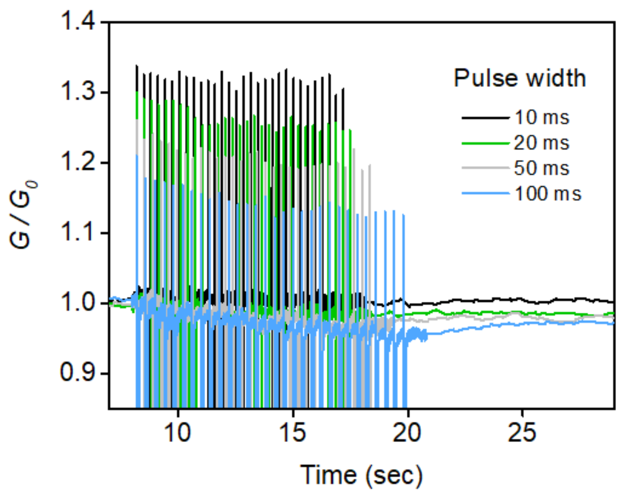

Figure S14. Short-term and long-term plasticity at various pulse widths. Thirty consecutive V_G pulses applied at pulse widths of 10, 20, 50 and 100 ms (V_G = 0.5 V, V_D = -0.2 V, pulse interval 300 ms, W/L = 10 μm/10 μm).

Long-term potentiation

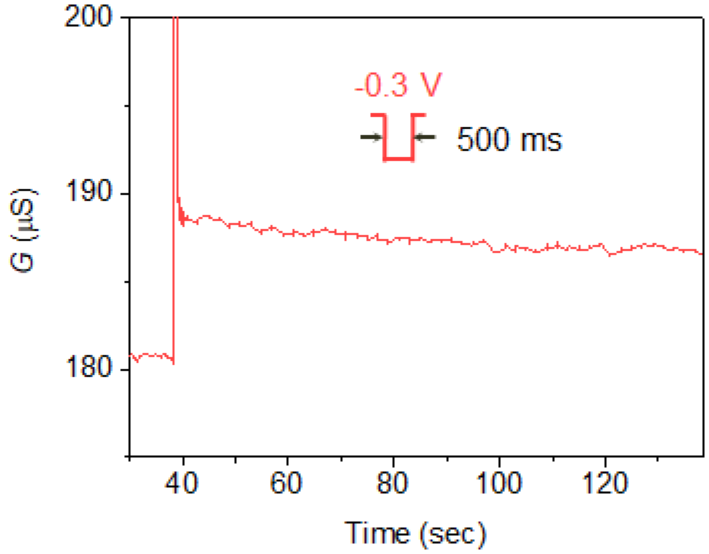

Figure S15. Long-term potentiation. LTP measured for 100 s after a V_G pulse (V_D = -0.2 V, *W/L* = 80 µm/10 µm).

Supplementary Tables

Table S1. Summary of energy consumption in various devices.

Reference	Channel material	Electrolyte	Active ions	Energy consumption
[5]	p-Si	$RbAg_4I_5$/MEH-PPV	Ag^+	10 pJ
[6]	IZO	SiO_2	H^+	45 pJ
[7]	CNT	PEG	H^+	7.5 pJ
[8]	ZnO_x	Ta_2O_5	$O2^-$	35 pJ
[9]	WSe_2	$LiClO_4$/PEO	Li^+	30 fJ
[10]	PEO/P_3HT	Ion gel		1.23 fJ
[11]	a-MoO_3	Ionic liquid	H^+	9.6 pJ
[12]	PEDOT:PSS/PEI	KCl	H^+	10 pJ
[13]	a-MoO_3	$LiClO_4$/PEO	Li^+	0.16 pJ
[14]	Graphene	$LiClO_4$/PEO	Li^+	500 fJ
[15]	MoS_2	PVA		23.6 pJ

Supplementary References

[1] G. Beamson, D. Briggs, *The Scienta ESCA 300 Database, Wiley, Chichester, UK*, **1992**.

[2] S. K. M. Jonsson, J. Birgerson, X. Crispin, G. Greczynski, W. Osikowicz, A. W. D. van der Gon, W.R. Salaneck, M. Fahlman, *Synthetic Metals* **2003**, 139, 1.

[3] X. Cristpin, X. Crispin, S. Marciniak, W. Osikowicz, G. Zotti, A. W. D. van der Gon, F. Louwet, M. Fahlman, L. Groenendaal, F. De Schryver, W. R. Salaneck, *J. Polym. Sci., Part B: Polym. Phys.* **2003**, 41, 2561.

[4] J. Rivnay, P. Leleux, M. Ferro, M. Sessolo, A. Williamson, D. A. Koutsouras, D. Khodagholy, M. Ramuz, X. Strakosas, R. M. Owens, C. Benar, J. M. Badier, C. Bernard, G. G. Malliaras, *Sci. Adv.* **2015**, 1, 4, e1400251.

[5] Q. Lai, L. Zhang, Z. Li, W. F. Stickle, R. S. Williams, Y. Chen, *Adv. Mater.* **2010**, 22, 2448.

[6] L. Q. Zhu, C. J. Wan, L. Q. Guo, Y. Shi, Q. Wan, *Nat. Commun.* **2014**, 5, 3158.

[7] K. Kim, C.- L. Chen, Q. Truong, A. M. Shen, Y. Chen, *Adv. Mater.* **2013**, 25, 1693.

[8] P. B. Pillai, M. M. De Souza, *ACS Appl. Mater. Interfaces* **2017**, 9, 1609.

[9] J. Zhu, Y. Yang, R. Jia, Z. Liang, W. Zhu, Z. Ur Rehman, L. Bao , X. Zhang, Y. Cai, L. Song, R. Huang, *Adv. Mater.* **2018**, 30, 1800195.

[10] W. Xu, S.-Y. Min, H. Hwang, T.-W. Lee, *Sci. Adv.* **2016**, 2, e1501326.

[11] C. S. Yang, D. S. Shang, N. Liu, G. Shi, X. Shen, R. C. Yu, Y. Q. Li, Y. Sun, *Adv. Mater.* **2017**, 29, 1700906.

[12] Y. van de Burgt, E. Lubberman, E. J. Fuller, S. T. Keene, G. C. Faria, S. Agarwal, M. J. Marinella, A. A. Talin, A. Salleo, *Nature Mater.* **2017**, 16, 414.

[13] C. S. Yang, C.- S. Yang, D.- S. Shang, N. Liu, E. J. Fuller , S. Agrawal, A. Alec Talin, Y.- Q. Li, B.- G. Shen, Y. Sun, *Adv. Funct. Mater.* **2018**, 28, 1804170.

[14] M. T. Sharbati, Y. Du, J. Torres, N. D. Ardolino, M. Yun, F. Xiong, *Adv. Mater.* **2018**, 30, 1802353.

[15] J. Jiang, J. Guo, X. Wan, Y. Yang, H. Xie, D. Niu, J. Yang, J. He, Y. Gao, Q. Wan, *Small* **2017**, 13, 1700933.

[16] D.A. Bernards, G.G. Malliaras, *Adv. Funct. Mater.* **2007**, 17, 3538.

[17] J. T. Friedlein, Mary J. Donahue, S. E. Shaheen, G. G. Malliaras, R. R. McLeod, *Adv. Mater.* **2016**, 28, 8398.

[18] M. A. Mukhamedyarov, S. N. Grishin, A. L. Zefirov, A. Palotas, P. Archiv, *Eur. J. Physiol.* **2009**, 458, 563.

[19] Y. Kim, A. Chortos, W. Xu, Y. Liu, J. Y. Oh, D. Son, J. Kang, A. M. Foudeh, C. Zhu, Y. Lee, S. Niu, J. Liu, R. Pfattner, Z. Bao, T.-W. Lee, *Science* **2018**, 360, 998.

[20] G. D. Spyropoulos, J. N. Gelinas, D. Khodagholy, *Sci. Adv.* **2019**, 5, eaau7378.
